# Evaluating disparities in counseling and resources for chemotherapy-induced hair loss: qualitative analysis of a survey-based approach

**DOI:** 10.1007/s00520-026-11377-4

**Published:** 2026-10-22

**Authors:** Lily Kaufman, Aliza Khuhro, Lilia Valentic, Randall Harris, Philip Binkley, Brittany Dulmage

**Affiliations:** 1 The Ohio State University College of Medicine, Columbus, OH USA; 2 Division of Epidemiology, College of Public Health, The Ohio State University, Columbus, OH USA; 3 Department of Medicine, Division of Cardiology, The Ohio State University Wexner Medical Center, The Ohio State University, Columbus, OH USA; 4 Department of Dermatology, The Ohio State University Wexner Medical Center, 540 Officenter Place, Suite 240, Columbus, OH 43230 USA

**Keywords:** Chemotherapy-induced alopecia, Chemotherapy-induced hair loss, Scalp cooling therapy, Alopecia treatment, Disparities in counseling and resource access

## Abstract

**Purpose:**

Chemotherapy-induced alopecia (CIA) is a distressing side effect of cancer treatment that significantly impacts patients’ quality of life. Despite the availability of interventions such as scalp cooling and topical treatments, many patients report inadequate counseling and limited access to these resources. This study aimed to evaluate disparities in CIA-related counseling and resource access, with particular attention to how socioeconomic and demographic factors shape the patient experience.

**Methods:**

We conducted a mixed-methods, cross-sectional survey study of 101 adults who had received chemotherapy and experienced hair loss. Participants were recruited through online cancer support networks. Thematic analysis was performed on open-ended responses to identify key patient-reported themes.

**Results:**

Thematic analysis revealed 11 key patient-identified themes, including emotional distress, provider influence, financial barriers, and a strong desire for autonomy in decision-making.

**Conclusion:**

This study highlights substantial gaps in CIA-related counseling and access, with disparities influenced by employment, income, and education. Early, empathetic, and comprehensive counseling plays a pivotal role in patient satisfaction and access to care. Standardizing CIA education and improving resource availability may help reduce inequities and improve patient outcomes.

**Supplementary information:**

The online version contains supplementary material available at https://doi.org/10.1007/s00520-026-11377-4.

## Introduction and background

Chemotherapy-induced hair loss, also referred to as chemotherapy-induced alopecia (CIA), is one of the most visible and emotionally distressing side effects of cancer treatment. Prior research has documented associations between hair loss and reduced quality of life, body image concerns, and psychosocial distress, underscoring the clinical importance of addressing CIA as part of comprehensive cancer care [1–6]. Beyond physical appearance, hair loss can disrupt patients’ sense of privacy and social identity, serving as a visible marker of illness that persists throughout treatment and recovery [7, 8]. As a result, CIA has been increasingly acknowledged as an important component of supportive oncology care rather than a purely cosmetic concern [1, 2]. In recent years, a range of strategies to prevent or manage CIA, including scalp cooling, wigs, and hair regrowth treatments, have become increasingly available to patients undergoing chemotherapy [9, 10].

Clinical guidelines and expert recommendations emphasize the importance of preparing patients for hair loss and providing anticipatory guidance throughout cancer treatment. Proposed frameworks for CIA counseling highlight the need to address expectations prior to treatment, provide support during active hair loss, assist with coping strategies, and offer guidance regarding regrowth [11–16]. Prior studies suggest that timely, clear, and empathetic communication about hair loss may reduce psychological distress and improve patient satisfaction [17–21]; however, the content, consistency, and perceived adequacy of such counseling vary widely across clinical settings.

Despite growing attention to the psychosocial impact of CIA and the availability of supportive interventions, patients’ experiences with counseling, information provision, and access to supportive resources are not well characterized and may vary substantially across individuals and care settings, and many patients do not receive adequate counseling about these resources [9, 10, 22, 23]. Much of the existing literature evaluating CIA-related counseling interventions has focused on clinical outcomes, feasibility, or use of interventions, with comparatively less emphasis on how patients learn about management and treatment options, how they experience the counseling process, or how emotional, practical, and contextual factors shape decision-making and satisfaction with care in real-world settings [1, 2, 9, 24, 25]. This disparity in access and communication is believed to be influenced by social determinants of health such as level of education, income, insurance status, and geographic location [26]. However, disparities in counseling and supportive care are often inferred from patterns of utilization or access rather than directly examined through patient narratives [10, 23, 26]. Consequently, limited research has explored how patients themselves perceive and navigate counseling, information gaps, and barriers related to CIA management. Qualitative approaches are well-suited to address this gap by centering the patient voice and capturing aspects of experience that are not readily measured through quantitative methods [27]. Accordingly, this study qualitatively explores cancer patients’ experiences with CIA using open-ended survey questions.

## Methods

### Study design and participants

This mixed-methods survey study used an observational, cross-sectional design to explore cancer patients’ experiences with chemotherapy-induced hair loss, access to preventive strategies such as scalp cooling, and perceived adequacy of counseling and support. Although both qualitative and quantitative data were collected, the present manuscript focuses exclusively on qualitative findings; quantitative results are reported in a companion manuscript. Eligible participants included adult individuals (at least 18 years old) who had received chemotherapy and experienced hair loss. Participants were recruited through US-based online cancer and chemotherapy support communities, including Facebook groups and the American Cancer Society Cancer Survivors Network; specific recruitment sources are provided in Appendix 1. Participation was not geographically restricted. The anonymous survey was advertised to adults who had received chemotherapy and experienced hair loss, and participant identity and medical history were not independently verified. A total of 101 individuals participated in the survey study.

### Survey instrument

The survey was administered using REDCap (Research Electronic Data Capture), a secure, web-based platform. The survey was open from January to May 2025. The study used a structured online survey comprising both closed- and open-ended questions; the present analysis focuses on responses to open-ended items. Open-ended questions invited participants to elaborate on their experiences with hair loss prevention, counseling, regrowth, and associated challenges. The list of open-ended questions can be accessed in Appendix 2.

### Qualitative analysis

Open-ended responses were analyzed using an inductive qualitative content analysis approach [28]. Survey data were exported from REDCap to Microsoft Excel, and open-ended responses were subsequently compiled in Microsoft Word for manual analysis. The primary investigator read all responses to become familiar with the data and identify recurring concepts. Conceptually similar responses were grouped and iteratively refined into broader categories and themes derived from the data. Representative quotations were selected to illustrate the resulting themes.

## Results

### Participant characteristics

A total of 101 participants completed the survey. Most participants had completed at least a bachelor’s degree (74.3%), and 56.4% were employed either full time or part time. Among participants who reported household income (*n* = 99), 57.6% reported an annual household income of $100,000 or greater. Age, gender, race/ethnicity, cancer type, and geographic residence were not collected.

### Qualitative results

Open-ended survey responses revealed 11 distinct themes reflecting patients’ experiences with chemotherapy-induced hair loss, counseling, and access to supportive resources. These themes are described below with illustrative quotes.

1. Information gaps and counseling deficits.
  Many patients reported inconsistent or absent counseling around hair loss, with limited details on scalp cooling, regrowth, or full-body hair changes. This included missed opportunities for proactive discussion and misleading or incorrect provider assumptions.
  “I didn’t receive any information from my oncologist.”
  “There was pretty much a lack of access to information, and negative attitudes in general about it.”
  “No doctors talk about body-wise hair loss… The bloody nose… from not having any nasal hair has been very difficult to deal with.”
  “The nurses don’t know how to apply the cold cap. You need to know how to do it yourself.”
  “I was falsely reassured that my hair probably wouldn’t fall out. She was wrong.”
2. Emotional and psychosocial impact.
  Hair loss was emotionally distressing for many, tied to identity, self-image, and mental health. Patients expressed that hair loss became a distressing visible sign of their illness. Patients also valued emotional validation and support but often found this aspect neglected by care teams.
  “I felt really self-conscious about losing my hair. I didn't want to look sick because I didn't feel sick.”
  “Because of the emotional effects of losing your hair… I think it's critical for real support to be offered through counseling.”
  “I wanted to keep my hair so I didn’t look sick at work.”
  “I had cancer as a child and lost my hair and wore a wig. I have more PTSD from [losing my hair] than the cancer and treatment. [I] was very worried about losing my hair. I wanted to present myself as ‘normal’ to the outside world even if I was going through treatment.”
  “I wish my providers had been more compassionate about the impact of hair loss.”
3. Provider influence: support, discouragement, or dismissal.
  Provider attitudes played a pivotal role—some empowered patients with resources and encouragement, while others dismissed concerns or discouraged preventive measures, affecting patient decisions and satisfaction.
  “My oncologist told me I would most likely lose all of my hair, even with cold capping. She was wrong.”
  “Information available, support from the cold capping company, a willing partner to help with the capping process, and support from oncologist.”
  “Provider did not recommend and staff did not encourage.”
  “My oncologist never heard of [cold capping], yet I read numerous messages… of women who used it with good results.”
  “Oncologist said nothing would work.”
  “It was not recommended by my oncologist.”
4. Financial and insurance barriers.
  High out-of-pocket costs, lack of insurance coverage, and the need for grant applications or self-funding were common obstacles to both prevention (e.g., scalp cooling), management (e.g., wigs), and regrowth treatments (e.g., minoxidil).
  “The cost of Penguin cap [was] too expensive and too much in the process of using.”
  “Cold capping was offered to me for free. If I had had to pay for it, I don’t know if I could’ve afforded it.”
  “I wish it had been covered by insurance.”
  “I was Medicaid pending when I started treatment.”
  “My insurance requires preauthorization for a wig, which I didn’t know before purchasing one.”
5. Logistical burdens and institutional limitations.
  Scalp cooling often required extensive coordination—dry ice, caregiver help, and long infusion times—and many centers lacked equipment, training, or support staff, making it difficult to implement effectively.
  “Professional systems are not available at my treatment center… then I’d still have to trudge in 60 pounds or so of dry ice each time.”
  “I wish my facility had capping machines so we didn’t have to take the machine back and forth.”
  “The process was exhausting, especially the post-infusion cooling.”
  “Nurses didn’t really know how to use the machine and seemed to be burdened.”
  “The self caps don’t stay cold long enough.”
6. Scalp cooling effectiveness and treatment experience.
  Patients reported mixed outcomes with scalp cooling, ranging from successful partial retention to complete failure. Some felt unprepared for its limitations or side effects, while others would still choose it again despite challenges.
  “I cold capped on my own and still lost a lot of hair, did not lose all hair.”
  “I wish I would’ve been given more information about how little hair you actually retain while cold capping.”
  “Session 1 without cold capping - hair fell out in handfuls. Session 2 using Paxman… hair also fell out.”
  “I went through it all, lost some hair but not all — it was worth it to me. I’d do it again even though it was hard.”
  “Was hopeful it would help save my hair. Infusion center staff very helpful. No barriers.”
7. Peer and community influence.
  Patients were often guided by experiences shared through social media, support groups, friends, or family. These non-medical sources frequently shaped decision-making more than formal counseling did.
  “My sister cold capped through chemo and saved a lot of her hair so I decided to try it.”
  “Penguin was highly rated on web and I had heard a story about it on the news several years ago.”
  “Social media reviews gave me hope in the cold cap being successful.”
  “The Paxman Facebook support group—I got lots of info from there.”
  “I'm thankful I had a friend who had capped that gave me specific advice.”
8. Cultural, personal, and family considerations.
  Decisions around hair loss were influenced by cultural identity, personal values, privacy concerns, and a desire to shield children or colleagues from visible signs of illness.
  “Being Native American, my hair is very important to me. I wanted to do anything I could to keep it.”
  “I wanted to maintain my privacy… I have little kids. They are in the heavy questioning phase.”
  “I didn’t want to be treated differently by coworkers, patients, or strangers.”
  “If I had a chance to look like myself I wanted to.”
  “Used cool cap so not have to look like a cancer patient and answer awkward questions from clients.”
9. Treatment side effects and cold intolerance.
  Physical discomfort—especially from cold exposure—was a key deterrent. Participants described treatment duration, cold sensitivity, anticipated discomfort, and concerns about restrictions associated with scalp cooling.
  “My treatments are 6 hours, I couldn’t sit with ice on my head that long.”
  “Because I hate cold, it wasn’t guaranteed to work… I wanted to continue working out.”
  “Most of it was cost, also already sensitive to cold.”
  “Not worth the discomfort.”
  “Couldn’t tolerate the cold and didn’t want to feel miserable during treatment.”
10. Regrowth strategies and post-treatment challenges.
  Patients sought guidance on regrowth timelines and methods, with some turning to products like minoxidil. Those who lacked information expressed frustration, and some faced ongoing hair changes or unmet expectations.
  “I found that minoxidil made a difference to regrowth definitely. I have a long relationship with my dermatologist already.”
  “[I wish I had] more information, more suggestions and recommendations for products/types of products to use or to stay away from […] What to expect during regrowth, recommend products or natural remedies to use to help regrowth […] [A]t the very least have a conversation about it and encourage people that there are lots of options out there.”
  “I am hoping to find regrowth options now that I am done with chemo.”
  “I will take any and all suggestions for regrowth.”
  “I bought Bondi Boost to encourage hair growth… that was good for my self-esteem.”
11. Desire for greater autonomy and choice.
  Patients emphasized wanting comprehensive information and the freedom to decide—even if they chose not to pursue interventions. Being denied options or agency often led to regret or dissatisfaction.
  “[I want] all options, then let me decide.”
  “Even if someone can’t afford it, at least they would have a choice.”
  “I chose to do it on my own and I told them they need to at least offer an option.”
  “Patients should be counseled before starting chemo.”
  “Oncology needs to at least offer options.”

**Fig. 1:**
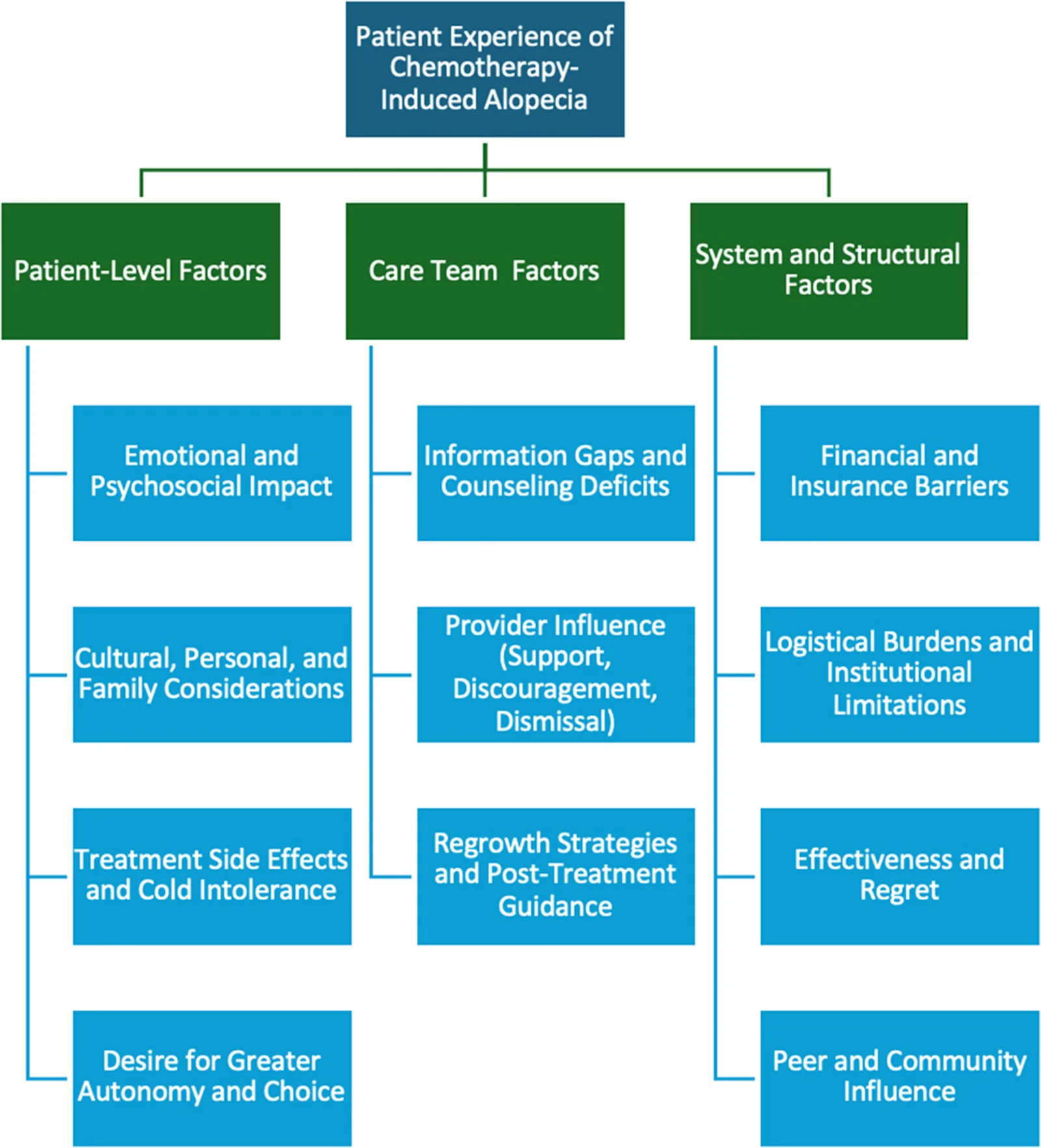
Multilevel factors influencing patient experiences of chemotherapy-induced alopecia

Figure 1 illustrates the relationships among the major themes identified in the qualitative analysis.

## Discussion

This qualitative analysis provides insight into how patients experience CIA and navigate prevention, regrowth, and support within real-world cancer care settings. Across themes, patient narratives revealed that CIA was rarely experienced as a minor or cosmetic side effect; instead, it was deeply intertwined with identity, emotional well-being, privacy, and perceived normalcy during treatment. Participants consistently described gaps between the clinical acknowledgement of hair loss and the level of guidance, empathy, and practical support they desired.

A central finding was the frequent perceived inadequacy of counseling surrounding CIA, extending beyond whether CIA was mentioned at all to how it was discussed. Many patients described receiving vague, incomplete, or potentially inaccurate information from healthcare providers, particularly regarding the availability and expected effectiveness of scalp cooling and options for hair regrowth. In the absence of comprehensive counseling, participants frequently sought information independently through online resources, social media communities, friends, and other patients with cancer. Although patients often described these peer and online sources as helpful, reliance on informal sources may result in variability in the quality and consistency of information available to patients. Informational gaps often left patients feeling unprepared and unsupported, contributing to distress, regret, and mistrust. Importantly, dissatisfaction was not limited to patients who lacked access to interventions. Even those who pursued scalp cooling or regrowth strategies described frustration when counseling failed to set realistic expectations or address the emotional burden of CIA. These findings align with existing patient studies regarding counseling for scalp cooling, supporting inadequate counseling as a source of significant psychosocial distress [29, 30]. It also suggests that inadequate counseling may represent a systemic gap rather than isolated incidences of inadequate communication, possibly driven by providers not having sufficient education or training on scalp cooling to provide sufficient counseling [30]. Therefore, emphasizing high-quality counseling may represent a strategy to mitigate the psychosocial burden and informational gaps that patients may face.

Provider attitudes emerged as a powerful influence on patient experiences. Some participants recalled clinicians who validated their concerns and offered resources, while others encountered dismissal, discouragement, or perceived gatekeeping. These interactions shaped not only access to preventive strategies but also patients’ sense of agency. Many respondents emphasized that the ability to choose, regardless of whether they ultimately pursued an intervention, was critical to their satisfaction. These differences in provider attitudes could reflect personal opinions on scalp cooling. A survey study of oncologists and dermatologists (*n* = 103) found significant differences in how CIA was viewed. Dermatologists were more likely to support pre-chemotherapy dermatologic assessments and advice regarding CIA, while oncologists were more likely to support CIA as a manageable side effect that could be treated using wigs and providing psychosocial support [31]. Another study of oncologists (*n* = 155) found that 67.1% of providers felt that all patients should be offered scalp cooling therapy, but only 26.3% report regularly initiating conversations about scalp cooling therapy and only 36% felt that scalp cooling benefits were worth the effort and cost involved [32]. Being denied information or feeling judged for prioritizing hair preservation often led to regret and disengagement, highlighting the importance of shared decision-making in supportive cancer care.

Structural and logistical barriers further complicated patients’ efforts to manage CIA. High costs, insurance limitations, institutional unpreparedness or lack of resources, and the physical burden of scalp cooling created obstacles even for patients motivated to pursue hair loss prevention or management. Scalp cooling frequently requires that patients self-pay due to lack of insurance coverage. Cost, structural, and logistical barriers have been recognized as predictors influencing the initiation of scalp cooling therapy discussions by oncologists [33]. Notably, participants frequently described relying on peer networks, social media groups, or friends with prior experience to fill gaps left by the healthcare system. A survey study of women in Facebook groups created for scalp cooling therapy users (*n* = 219) found that key motivations for utilizing social media in scalp cooling therapy included to gain support from others going through a similar experience (88.1% of participants), to receive scalp cooling instructions (82.2%), and to learn about the scalp cooling therapy outcomes of other patients (69.9%) [34]. While these communities provided valuable practical and emotional support, their prominence also underscores systemic shortcomings in formal counseling and institutional resources. As described by our survey respondents, information and guidance shared in online support groups is shared by word-of-mouth and typically anecdotal rather than evidence-based.

CIA was repeatedly framed as a threat to identity, privacy, and emotional stability. Patients described efforts to avoid “looking sick,” protect children or colleagues from visible signs of illness, and maintain continuity in work and social roles. The severity of the emotional burden patients may experience due to CIA should not be underestimated. In a study of 179 patients undergoing chemotherapy, 56.4% reported that hair loss was the worst side effect, with 11.7% finding it worse than the disease itself and 16.2% reporting that hair loss would have made them stop chemotherapy if it was not a life-saving treatment [35]. Another study found that patient experiencing higher distress due to CIA were also more likely to report poorer body image, emotional and social functioning, and depression compared to patients with less distress from CIA [2]. For some, prior traumatic experiences with cancer-related hair loss amplified distress during treatment. These narratives suggest that CIA counseling should not be treated as an ancillary discussion but as an integral component of psychosocial oncology care.

These findings underscore the need for standardized, evidence-based counseling about CIA that begins before chemotherapy and supports informed, preference-sensitive decision-making. Such counseling could be incorporated into routine pre-chemotherapy education and supported by written or online resources developed or endorsed by oncology professional organizations. Standardized resources should address the anticipated extent and course of hair loss, available preventive and management strategies, expected effectiveness and limitations of scalp cooling, potential adverse effects and logistical considerations, financial and insurance considerations, wigs and other cosmetic resources, and expectations and management options for post-chemotherapy hair regrowth. Providing consistent information while allowing counseling to be tailored to individual priorities may help reduce informational disparities and support patient autonomy. These findings also indicate that improving care for CIA requires more than expanding access to preventive technologies. Meaningful improvement depends on empathetic, anticipatory communication, provider education that encompasses both technical and emotional aspects of hair loss, and systems that support patient autonomy and reduce financial and logistical barriers to care. Standardized counseling that addresses prevention, limitations, regrowth, and emotional impact while acknowledging individual values and circumstances may help reduce distress and improve patient experience, regardless of whether patients pursue specific interventions.

This study’s strengths lie in its focus on patient narratives, which highlight dimensions of CIA that are not captured through quantitative measures alone. Analysis of open-ended responses allowed for a nuanced exploration of counseling experiences, emotional responses, decision-making processes, and system-level barriers. By centering patient voices, this study provides insight into how care delivery is experienced and where opportunities for improvement exist.

Several limitations should be considered. Participants were self-selected and primarily recruited through online cancer support communities, which may overrepresent individuals who are more engaged, resource-seeking, or motivated to share their experiences. Some online communities focused specifically on scalp cooling, which may overrepresent individuals who are particularly interested in CIA prevention. Although recruitment was conducted through U.S.-based online communities, participation was not geographically restricted and participant country of residence was not verified, limiting conclusions about the geographic context of these experiences. Because participation was anonymous and participant eligibility was not independently verified, respondent identity and treatment history could not be confirmed. Responses were retrospective and self-reported, introducing potential recall bias, particularly given the emotional intensity of cancer treatment. Additionally, the survey format limited the ability to probe responses or explore themes in greater depth, and certain demographic and contextual factors that may shape experiences were not assessed. Age, gender, race/ethnicity, cancer type, and geographic residence data were not collected, limiting assessment of how these characteristics may have shaped participants’ experiences. Finally, qualitative coding and theme development were conducted by a single investigator, which may have introduced interpretive bias. Future qualitative studies incorporating interviews and more diverse populations may help deepen understanding and inform targeted interventions.

## Conclusion

CIA remains a significant and often inadequately addressed aspect of cancer care. Participants described gaps in counseling, variable access to preventive and management strategies, financial and logistical barriers, and unmet needs related to hair regrowth and psychosocial support. Standardized, evidence-based CIA education incorporated into routine pre-chemotherapy counseling represents an important starting point for improving care by ensuring that patients receive consistent information about anticipated hair loss and available prevention and management options. Such education should be paired with individualized, preference-sensitive counseling and efforts to improve access to supportive resources. Addressing these gaps may better support informed decision-making, patient autonomy, and the psychosocial well-being of individuals experiencing CIA.

## Supplementary information

Below is the link to the electronic supplementary material.

ESM 1 (DOCX 14.6 KB)

ESM 2 (DOCX 20.0 KB)

## Data Availability

No datasets were generated or analysed during the current study.
