# Supplementary material for: Evaluating disparities in counseling and resources for chemotherapy-induced hair loss: qualitative analysis of a survey-based approach
Source: Support Care Cancer. 2026 Oct 22;34(11):1143. doi: 10.1007/s00520-026-11377-4 (PMC13690825; doi:10.1007/s00520-026-11377-4)
Supplement: Supplementary file 1 [file 520_2026_11377_MOESM1_ESM.docx]

**Appendix 1. Online Recruitment Sources**

| Recruitment Source | Platform |
| --- | --- |
| Chemo Cold Cappers Support Group | Facebook support group |
| Chemotherapy Support Group | Facebook support group |
| Tips for Getting Through Chemo | Facebook support group |
| Paxman Scalp Cooling Group | Facebook support group |
| Cancer Survivors and Supporters | Facebook support group |
| Chemo Cold Caps | Facebook support group |
| Cancer Support | Facebook support group |
| Breast Cancer Support Group | Facebook support group |
| Cancer Survivors Network | American Cancer Society |

**Note.** Recruitment was conducted through U.S.-based online support communities. Participation was not geographically restricted, and participant country of residence was not verified due to the anonymous survey design. Recruitment sources are listed as they existed during the study recruitment period (January-May 2025). The Cancer Survivors Network was an online discussion community operated by the American Cancer Society at the time of recruitment.
