# Supplementary material for: Evaluating disparities in counseling and resources for chemotherapy-induced hair loss: qualitative analysis of a survey-based approach
Source: Support Care Cancer. 2026 Oct 22;34(11):1143. doi: 10.1007/s00520-026-11377-4 (PMC13690825; doi:10.1007/s00520-026-11377-4)
Supplement: Supplementary file 2 [file 520_2026_11377_MOESM2_ESM.docx]

**Appendix 2. Open-Ended Survey Questions Related to Counseling and Resource Access for Chemotherapy-Induced Alopecia.**

*The following survey questions generated free-text responses analyzed in this study. Free-text data included responses to open-ended questions and free-text responses provided under “Other (please specify)” options within multiple-choice items. The quantitative results of the multiple-choice survey items have been reported in a separate manuscript. Multiple-choice response options are included below for context.*

**Counseling About Hair Loss, Scalp Cooling, or Regrowth Options**

If you were counseled about hair loss prevention or regrowth, what information was most helpful, and what do you feel was missing?

**Access to Scalp Cooling**

If you used scalp cooling, please describe your experience, including any challenges or benefits you encountered.

**Referrals and Hair Regrowth Options**

What types of hair regrowth options were suggested by your health care provider?

a. Over-the-counter treatments (e.g., minoxidil or Rogaine)
b. Prescription medications
c. Vitamins or supplements
d. Other (please specify):

If you were referred to a dermatologist or provided hair regrowth options, how did these influence your recovery experience?

**Barriers and Perceived Gaps in Resources**

If you were aware of hair loss prevention options but did not pursue them, what were the reasons?

a. Cost or financial concerns
b. Lack of availability in my area
c. Provider did not recommend it
d. I chose not to pursue it for personal reasons
e. Other (please specify):

If you decided not to pursue certain options for hair loss prevention or management, please share why and describe any challenges you face in making this decision.

**Patient Experience and Perceived Gaps in Care**

What influenced your decision to use or not use specific treatments for hair loss during chemotherapy? Please describe your thought process, preferences, and any barriers you encountered.

What support or resources related to hair loss do you wish had been offered to you?

What barriers, if any, did you face when trying to access treatments or information about managing hair loss?

Please describe any suggestions you have for improving care and support for patients experiencing chemotherapy-induced hair loss.
